# Recent evidence from omic analysis for redox signalling and mitochondrial oxidative stress in COPD

**DOI:** 10.1186/s12950-022-00308-9

**Published:** 2022-07-11

**Authors:** Sharon Mumby, Ian M Adcock

**Affiliations:** Airways Disease National Heart & Lung Institute Imperial College London, Guy Scadding Building Dovehouse Street, SW3 6LY London, UK

**Keywords:** COPD, Transcriptomics, Mitochondria, Oxidative stress

## Abstract

COPD is driven by exogenous and endogenous oxidative stress derived from inhaled cigarette smoke, air pollution and reactive oxygen species from dysregulated mitochondria in activated inflammatory cells within the airway and lung. This is compounded by the loss in antioxidant defences including FOXO and NRF2 and other antioxidant transcription factors together with various key enzymes that attenuate oxidant effects. Oxidative stress enhances inflammation; airway remodelling including fibrosis and emphysema; post-translational protein modifications leading to autoantibody generation; DNA damage and cellular senescence. Recent studies using various omics technologies in the airways, lungs and blood of COPD patients has emphasised the importance of oxidative stress, particularly that derived from dysfunctional mitochondria in COPD and its role in immunity, inflammation, mucosal barrier function and infection. Therapeutic interventions targeting oxidative stress should overcome the deleterious pathologic effects of COPD if targeted to the lung. We require novel, more efficacious antioxidant COPD treatments among which mitochondria-targeted antioxidants and Nrf2 activators are promising.

## Introduction

Chronic exposure to inhaled irritants including cigarette or biomass smoke and/or environmental pollutants are the principle causes of chronic obstructive pulmonary disease (COPD). The Global Burden of Disease analysis has recently reported that COPD caused 3.2 m deaths annually and also has a considerable health-care and societal burden globally [1]. These numbers are likely to rise as people are now living longer. Currently, 2 billion people are smokers or are exposed to second-hand smoke, over 2 billion people are affected by biomass fuel use and ∼1 billion of the population are exposed to the detrimental consequences of outdoor air pollution [1]. As a consequence, in 2019 there were almost 550 m people globally with a chronic respiratory disease, mostly due to COPD, with only cardiovascular disease (CVD) and cancer causing more deaths annually [1]. Across the EU, the annual health-care costs, including primary and inpatient health care costs, for chronic respiratory diseases was €380 billion in 2019 [1].

**Fig. 1:**
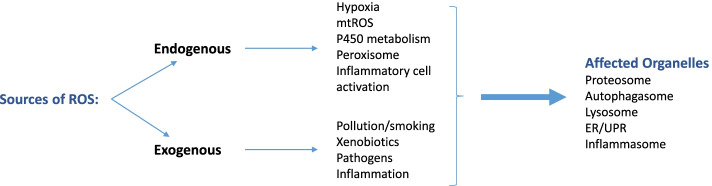
Sources of reactive oxidant species (ROS) within the lung. The high levels of ROS reported in the lung and airways of COPD subjects is derived from both exogenous and exogenous sources. Exogenous sources include cigarette smoking, environmental pollution, pathogens and inflammation. ROS is generated endogenously through mitochondria including mitochondrial ROS (mtROS), peroxisome activation, hypoxia and inflammation. ROS affects the function of many intracellular organelles such as proteasome, inflammasome, lysosome and the endoplasmic reticulum (ER) via the unfolded protein response (UPR) to elicit detrimental effects on cellular functions

**Fig. 2:**
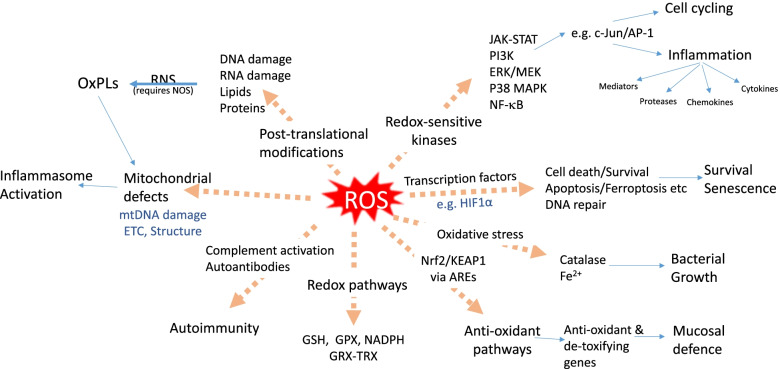
Molecular and cellular targets of reactive oxygen species (ROS) in the airways and lungs of COPD patients. ROS directly affects the activity and/or expression of redox-sensitive kinases, transcription factors, mitochondria, anti-oxidant pathways, iron (Fe) biology and innate immune systems such as complement and autoantibody production. Modulation of these processes promotes cell proliferation/survival and cellular senescence which is associated with enhanced inflammation. Enhanced oxidant pathways and reduced anti-oxidant activity affects mucosal defence against bacteria and viruses including reduced phagocytosis, whilst oxidative stress actions on catalase and Fe allow excess bacterial growth. ROS also causes post-translational modifications of DNA, RNA, lipids and proteins to affect cellular function and reveal neo-epitopes for auto-antibody induction. The generation of oxidised phospholipids (OxPLs) can further drive mitochondria dysregulation and activate the inflammasome. Abbreviations: AP-1: activator protein-1; ARE, anti-oxidant response element; ERK, extracellular signal-regulated kinase; ETC, electron transport chain; GSH, glutathione; GPX, glutathione peroxidase; GRX, glutaredoxins; HIF1α, hypoxia-Inducible Factor 1α; JAK-STAT, Janus kinase-signal transducer and activator of transcription; KEAP, Kelch-like ECH-associated protein; MEK, mitogen-activated extracellular signal-regulated kinase; mtDNA, mitochondrial DNA; NADPH, nicotinamide adenine dinucleotide phosphate; NF-κB, nuclear factor κB; NOS, nitric oxide synthase; Nrf2, Nuclear factor-erythroid factor 2-related factor 2; p38 MAPK, p38 mitogen activated protein kinase; PI3K, phosphoinositide 3-kinase; RNS, reactive nitrogen species; TRX, thioredoxins

COPD is considered a chronic immunoinflammatory disease of the airway, which drives severe airflow limitation due to subsequent remodelling of the small airways together with mucus hypersecretion and/or alveolar wall destruction or emphysema [2]. The inflammatory response in COPD is linked to the recruitment and activation of neutrophils and alveolar macrophages and dysregulation of structural cells such as epithelial cells with enhanced mucus production (bronchitis) and attenuation of ciliated epithelial cell function [2]. The release and function of the numerous inflammatory cytokines, chemokines, growth factors and other mediators that are elevated in COPD is exacerbated by the presence of endogenous and exogenous oxidative stress [2] (Fig. 1). There is increasing evidence for a role of oxidant stress-induced autoimmunity against modified self-antigens in COPD as well as the recognition of premature senescence occurring in the lungs and airways of COPD patients [3] (Fig. 2). Bacterial and viral infections result in acute exacerbations of COPD due to an altered innate immune system and this is a major cause of death in COPD [4]. The global initiative for chronic obstructive lung disease (GOLD) guidelines now assesses severity of COPD according to symptoms and the frequency of exacerbations [5]. Current therapies for COPD are directed towards palliative effects on lung function and none of the current therapeutic agents reverse inflammation and prevent disease progression [2].

The mechanisms that drive COPD pathogenesis are not well understood beyond the recognition of the key importance of oxidative stress, but the increasing understanding and identification of pathways involved in disease phenotypes may provide new therapeutic opportunities.

### Reactive oxygen species (ROS) and oxidative stress

Oxygen is essential for the supply of energy to eukaryotes but it also forms detrimental ROS and the related nitrogen species (RNS) following both enzymatic and non-enzymatic processes [6]. This leads to protein, lipid and DNA damage and must therefore be kept under tight regulatory control by the presence and activity of antioxidants located within cells and in the lung epithelial lining fluid [6]. External factors such as infection, air pollution or cigarette smoke exposure can overcome the local antioxidant capacity whilst internal sources of ROS include inflammatory cell activation and disease. Persistent ROS is associated with several airway diseases including asthma, COPD and lung cancer [6]. Indeed, it is recognised that COPD pathogenesis involves a disturbed balance between antioxidant defences and enhanced oxidative stress [7].

Glutathione is a major redox buffer that acts as an antioxidant defence mechanism to protect the lung from oxidative stress [8]. More recently, glutathione is recognized for its ability to induce S-glutathionylation which can change the structure and function of the target protein [8]. S-glutathionylation also allows the protein to be regenerated enzymatically as it protects them from irreversible oxidation. Glutathione S-transferases and glutaredoxins catalyze this process [8].

There are increased levels of ROS in COPD due to enhanced numbers of superoxide anions (O^−^ _2_), hydroxyl radicals (•OH), hydrogen peroxide (H_2_O_2_) and suppression of antioxidant and antiinflammatory gene expression. Key factors include heme oxygenase (HO)-1, glutathione peroxidase (GPx) and thiol metabolism-associated detoxifying enzymes (glutathione S-transferases, GSTs) together with antioxidant transcription factors including nuclear factor erythroid 2-related factor (Nrf)2 [7]. In addition, oxidative stress in the presence of nitric oxide (NO) results in the formation of various RNS including peroxynitrite (ONOO ^−^) which cause cell damage and disruption of biological processes by inducing protein and DNA nitration that impacts upon DNA damage/repair, mitochondrial respiration and inflammation. Myeloperoxidase (MPO) activation as well as H_2_O_2_ can also promote nitration of proteins following nitrite (NO2 -) oxidation [7] (Fig. 2).

COPD is often considered as a disease of premature lung ageing which itself is associated with abnormal responses to oxidative stress [9]. Age-related changes in cell quality control systems are linked particularly with a reduced ability to undertake redox and protein homeostasis. This age-related redox imbalance may act as an initial cellular ‘hit’ that induces cell adaptive stress-response pathways, increases oxidative stress with the resulting enhancement of lung injury leading to COPD [9]. Thus, a number of ‘secondary hits’ including smoking and environment-related pollution together with infections could the primed or dysregulated adaptive defence and repair pathways with further enhanced redox stress that results in the onset and progression of COPD [9].

### Mitochondrial ROS and COPD

Mitochondria are critical components of redox signaling and their aberrant function is linked to abnormal oxidative stress and metabolic dysfunction [10]. In addition to signaling by ROS, mitochondria regulate many cellular processes including cellular survival, control of anabolic and metabolic pathways and of innate immune signaling factors that are all altered in COPD [10]. Furthermore, in some patients with COPD, there is a hypoxic drive to their disease due to ventilation:perfusion mismatch which will also impact on mitochondrial function [11]. Hypoxia, which is sensed by the transcription factor HIF1α, results in further ROS production and oxidative damage, heightened inflammation and a metabolic switch towards a more glycolytic state [11]. The overall change in mitochondrial function and metabolic adaptation to an altered local microenvironment results in the production of distinct metabolic intermediates that can modulate the inflammatory response by acting as signaling molecules [11] (Fig. 2).

Enhanced levels of mitochondrial ROS (mtROS) as well as decreased mitochondrial membrane potential (ΔΨm) and mitochondrial superoxide dismutase (SOD2) levels were observed in bronchial biopsies from COPD patients [12]. ΔΨm in lung samples significantly correlated with forced expiratory volume in 1 s (FEV1, % predicted), 6-min walk test, lung carbon monoxide transfer factor (TLCOC % predicted) and maximum oxygen consumption. Increased total ROS and mtROS were found in the quadriceps muscle of the same patients with no effect on ΔΨm, SOD2 or levels of electron transport chain (ETC) complex proteins [12]. The data suggest that the mitochondrial changes observed in the quadriceps muscle are likely to result from spill over from the lung.

Monocyte-derived macrophages (MDMs) and alveolar macrophages from COPD patients are less effective at bacterial phagocytosis than cells from healthy control subjects [13]. This leads to impaired responses against exacerbation triggers in COPD and heightened airway inflammation in these subjects [14]. For example, COPD alveolar macrophages secrete excess proinflammatory mediators and proteases and express an altered pattern of surface and intracellular markers [15]. Phagocytosis of Haemophilus influenzae and Streptococcus pneumoniae by COPD cells, but not cells from healthy smokers or non-smokers, increased early mtROS levels and decreased ΔΨm [13] (Fig. 2). Furthermore, exogenous ROS decreased the phagocytic activity of control alveolar macrophages [13] suggesting that drugs that restore mitochondrial dysfunction may improve the defective phagocytic response seen in COPD macrophages. Indeed, MDMs exposed to cigarette smoke displayed inhibited bacterial phagocytosis via a reduction in alveolar macrophage cystic fibrosis transmembrane conductance regulator (CFTR) expression [16]. The effects of cigarette smoke on phagocytosis were attenuated by the free radical scavenger N-acetylcysteine [16].

Type II alveolar epithelial (ATII) cells from emphysematous subjects generate high levels of mitochondrial superoxide, exhibit mtDNA damage with associated mitochondrial dysfunction [17]. The degree of impaired mitochondrial fission/fusion as suggested by reduced expression of mitofusin 1 (MFN1), optic atrophy 1 (OPA1), Fission, Mitochondrial 1 (FIS1) and phosphorylated dynamin-related protein 1 (p-DRP1) correlated with level of emphysema [17]. An interesting study that compared the ATII like cell line A549 with mitochondria to those without mitochondria (A549-Rho-0), showed the loss of mitochondria resulted in enhanced pro-inflammatory mediator release, reduced epithelial repair functions and a loss in corticosteroid sensitivity [18] (Fig. 2). This latter effect appeared to be dependent upon glycolytic reprogramming and altered phosphoinositide-3-kinase (PI3K) activity [18]. This study highlights the importance of mitochondrial homeostasis on lung epithelial cell responses and how these may contribute to COPD pathogenesis.

Cigarette smoke-induced mitochondrial dysfunction (mtROS, oxidative phosphorylation or OXPHOS protein expression, structural changes and ΔΨm) in airway epithelial cells isolated from human lung can result in cellular senescence [19]. Cigarette smoke exposure also altered mitochondrial respiration as indicated by markers of oxygen consumption rate including maximum respiration, production of ATP and oxygen spare capacity) in BEAS-2B cells and NHBE cells [19]. Mechanistically, these changes were associated with reduced mitochondrial Rho GTPase 1 (MIRO1) and PTEN Induced Kinase 1 (PINK1) expression highlighting their potential role in the pathogenesis of COPD [19]. In primary human airway fibroblasts from COPD patients, the mitochondrial-associated senescence phenotype was reversed by pharmacologic induction of HO-1 [20]. HO-1 up-regulation in COPD cells enhanced the replicative capacity and attenuated the senescence and inflammatory capacity following restoration of ‘normal’ mitochondrial respiration, glycolysis and ATP levels and a reduction in the enhanced mtROS production and restored mitophagy [20].

The development of non-small cell lung carcinoma is increased in COPD patients and their disease has a worse prognosis [21]. Carcinogenesis is driven, at least in part, by abnormal mitochondrial function enhanced oxidative stress and the expression of phosphoglycerate mutase family member 5 (PGAM5), a mitophagy regulator, was highly expressed by alveolar macrophages from COPD patients and in malignant and pre-neoplastic epithelial cells [21]. Macrophage PGAM5 levels trended towards being greater at the periphery of the cancer in patients with COPD. Together these data suggest that PGAM5 expression is associated with patient mortality and that this may be linked to abnormal mitochondrial function in specific subsets of macrophages [21].

Cellular biosynthetic and redox pathways are influenced by changes in fatty acid oxidation (FAO) and glycolysis and these extensive metabolic changes play an important function in innate immunity in COPD [4]. COPD airway smooth muscle (ASM) cells possess an aberrant mitochondrial function and a specific metabolic phenotype that is associated with enhanced growth [22]. For example, their energy production is abnormal with enhanced generation of lactate, glutamine, fatty acids and amino acids compared to cells from healthy subjects under both stimulated and unstimulated conditions. In addition, FAO capacity was attenuated at baseline in COPD ASM cells which was restored by stimulation with transforming growth factor-β (TGFβ)/foetal calf serum [22]. This was accompanied by elevated flux through the pentose phosphate shunt and of nucleotide biosynthesis. Together, this suggests that differences in glycolysis, glutamine and fatty acid metabolism occur in COPD ASM cells resulting in increased biosynthesis and redox balance which switch the cellular phenotype towards supporting cell growth in COPD [22].

As an alternative to reversal of COPD-associated mitochondrial defects in airway smooth muscle cells by small molecule drugs, Li and colleagues used mesenchymal stem cells (MSCs) to deliver ‘healthy’ mitochondria to COPD cells and in vivo to ozone-exposed animals [23]. Culture of MSCs with airway smooth muscle cells attenuated cigarette smoke-induced increased mtROS and reduction in ΔΨm loss which was associated with apoptotic cell death [23]. In these experiments, transfer of healthy mitochondria from MSCs to smoke-exposed airway smooth muscle cells occurred via tunnelling nanotubes. These results were recapitulated in vivo in murine lungs where MSCs reduced ozone-induced mitochondrial dysfunction, inflammation and airway hyperresponsiveness. The authors indicated that an MSC-based therapeutic approach may be useful in COPD [23].

Mitochondrial dysfunction in COPD may be greater in females than males [24] particularly in COPD patients with an emphysema-predominant disease [25]. Using an integrated network interference approach to analyse transcriptomic datasets from COPD and healthy controls, it was possible to identify gene sets involved in mitochondrial function and energy metabolism as being sexually dimorphic [24]. This data is supported by a recent analysis of the Emphysema versus Airways Disease project (EvA) whereby in a large study of COPD patients (312 subjects) with transcriptomic data from bronchial brushings there was a difference in the number of differentially expressed genes in males (*n* = 40) and in females (*n* = 73) between healthy and COPD [25]. Male COPD patients particularly those with an emphysema phenotype expressed a signature of mitochondrial-encoded functional genes.

### Iron (Fe), oxidative stress and COPD

Disrupted iron homeostasis is linked to severity of stable COPD and during acute exacerbations of COPD (AECOPD) possibly as a result of iron regulatory protein (IRP)-2 polymorphisms and independent of anaemia [26]. In a small observational study of exhaled breath condensate there was an attenuated capacity to respond correctly to cigarette smoke-induced iron handling and excretion (production of redox active iron) in patients with COPD [27] (Fig. 2) with iron responsive element binding protein 2 (IREBP2) protein being raised in COPD lung [28]. The usual cigarette smoke-induced COPD-phenotype is not observed in mice deficient in IREBP2 due to prevention of mitochondrial dysfunction. Interestingly, mice fed a mitochondrial iron chelator also did not demonstrate the cigarette smoke-induced COPD-phenotype [28]. In addition, the expression of the iron regulatory peptide hepcidin is reduced in COPD and this is recapitulated in mice after cigarette smoke exposure [29]. Murine cigarette smoke-exposed alveolar macrophages and human alveolar macrophages from smokers also have elevated levels of ferroportin. This dysregulated hepcidin/ferroportin axis contributes to reduced phagocytosis of bacteria and an enhanced response to Streptococcus pneumoniae infection [29] (Fig. 2).

Bronchoalveolar lavage (BAL) fluid (BALF) levels of iron and ferritin were elevated in subjects from the SPIROMICS (Subpopulations and Intermediate Outcomes in COPD Study) study and were raised to a greater extent in COPD patients with more exacerbation [30]. However, BAL levels did not correlate with systemic iron markers [30]. Overall, enhanced iron retention in the airways and lungs of patients with COPD can contribute to the oxidative stress-induced cellular damage and microbial virulence [27]. Overall, there is a link between iron levels, oxidative stress and the host-airway microbiome [31, 32].

Both oxygen and iron (Fe) are important in the formation of ROS [33]. There were significantly lower serum levels of antioxidant carotenoids which regulate Fe levels in COPD subjects (*n* = 66) compared with healthy controls (*n* = 47) [33]. There was no significant difference in serum protein carbonylation (PC) but soluble transferrin receptor (sTfR) levels were elevated. sTfRs, which correlate inversely with Fe status, were associated negatively with PC suggesting that Fe levels are associated with enhanced oxidative stress [33].

Altered iron metabolism may also affect airway remodelling and immune functions in COPD. Ferroptosis, a necrotic form of programmed cell death involves phospholipid peroxidation together with Fenton reactions that are mediated by free iron [34]. Labile iron accumulation and enhanced lipid peroxidation was observed in both in vivo and in vitro cigarette smoke exposure models of COPD in the absence of apoptotic cell death. Ferrostatin-1, an inhibitor of ferroptosis, and GPx4 knockdown indicated the key role of ferroptosis in models of COPD pathogenesis [34].

### Unbiased omics analysis and oxidative stress markers in COPD

Analysis of oxidant/antioxidant defects in COPD are often derived from focussed assays but the advent of unbiased or semi-biased transcriptomic and proteomic platforms has enabled the detection of oxidative stress and of other key immune and inflammatory pathways that are present in COPD. A recent review has examined the utility and reliability of oxidative stress biomarkers in case control studies in COPD patients [35]. Most analyses have been conducted in the blood compartment as it is readily accessible and allows repeated testing. Exhaled breath is difficult to standardise whilst induced sputum is partially invasive and not produced by all subjects. In general, exhaled H_2_O_2_, 8-isoprostane, malondialdehyde, ethane and peroxynitrites are elevated in COPD compared to control subjects [35]. Samples from bronchial biopsies and bronchial brushings are limited due to the invasive nature of their collection [35] but protein carbonylation, lipid peroxidation and 3-nitrotyrosine levels are reported as upregulated in COPD biopsies [35].

### Lung tissue

RNA-sequencing analysis from lung tissue from 91 COPD cases and 182 matched healthy controls from the Genotype-Tissue Expression (GTEx) database identified 1359 differentially expressed genes (DEGs) with 707 genes being up-regulated and 602 being downregulated [36]. The results were validated in a separate cohort (108 healthy subjects and 219 COPD patients) with pathway analysis highlighting the importance of complement activation, dysregulated inflammation and extracellular matrix (ECM) organization in patients with COPD. 15 key central or hub genes were identified and were mostly involved in cell proliferation but included HIF1α which is involved in sensing oxygen levels, ROS production and oxidative damage and heightened inflammation [11]. These results were validated in a separate study whereby network analysis identified central molecular hubs or “switch genes” in COPD [37]. A COPD correlation network was found that had three modules: one contained up-regulated switch genes associated with the control of immune and inflammatory responses as well as hypoxia including BLNK, HIF1A, LY96, PRDX4, SYK and TIMP1; one containing up-regulated immune genes and another containing reduced expression of the genome-wide association study (GWAS)-identified genes AGER and CAVIN1 in COPD cases [37]. The immune genes within module 2 had not been previously associated with COPD by GWAS for example. Severe hyperoxia in new-born mice results in an emphysema phenotype in adulthood in the absence of oxidative stress and inflammation and indicates an important role for fragmented elastic fibres in adult emphysema [38].

In a separate study, transcriptomic analysis of lung tissue from 98 COPD patients and 91 controls [39] identified 2312 DEGs with dysregulation of pathways related to oxidative phosphorylation, chromatin modifications and protein catabolism [39]. In addition, transcriptomic analysis of lung tissue using a novel deconvolution process identified genes associated with mild-moderate COPD [40]. Protein-protein interaction subnetworks showed mitochondrial dysfunction and an aberrant immune response as being the pathways most dysregulated in these tissues [40].

DNA damage and repair pathways, often due to oxidative stress, are important in the onset and development of COPD [41] (Fig. 2). A focused analysis of 419 genes regulating DNA damage and repair mechanisms in lung samples from COPD patients from 3 independent cohorts identified 15 DEGs in severe disease [42]. Suppression of the nucleotide excision/repair pathway was most closely associated with increasing COPD severity [42].

Network analysis of lung transcriptomic data from 70 former smokers with COPD identified differences in gene profiles between subjects with bronchiolitis and emphysema [43]. Emphysema patients, but not subjects with bronchiolitis, were associated with enhanced B-cell homing and activation pathways and the expression of genes such as CXCL13, CCL19 and POU2AF1 correlated with emphysema severity. This was replicated in a separate cohort of predominantly emphysematous COPD subjects [43]. In contrast, RNA-sequence analysis of COPD lung tissue from 29 emphysematous COPD patients, 21 non-emphysematous COPD patients and 60 smokers without obstruction identified 1,226 DEGs in emphysematous COPD and 434 DEGs in non-emphysematous COPD compared to healthy smokers [44]. In the emphysematous COPD patients, the highest DEGs were ACER2 (up-regulated) and LMAN2L (down-regulated) whilst the cholinergic receptor muscarinic 3 (CHRM3, up-regulated) and histone deacetylase (HDAC)10 (down-regulated) were similarly represented in the COPD without emphysema group [44].

Although many smokers with normal lung function exhibit clear evidence of emphysematous changes they are not classified as having COPD [45]. An important study examining lung tissue from patients with emphysema but with normal lung function compared with COPD patients (*n* = 12 in each group) demonstrated no difference in inflammation, protease-anti-protease balance, oxidative stress (glutathione and SOD) and apoptosis between the groups when the degree of emphysema was accounted for [45]. This provides evidence that FEV1 alone is not optimal for the diagnosis of COPD and that emphysematous pathways are similar in smokers with or without lung obstruction [45].

Using previously published transcriptomic datasets for resected bronchial tissues from COPD patients with or without lung squamous cell carcinoma it was possible to show using Weighted Gene Co-expression Network Analysis (WGCNA) that both diseases shared pathogenic pathways including those closely related to oxidative stress, the immune response and infection [46].

Tandem mass tag labelled quantitative proteomics of lung tissue from COPD patients who were either frequent or infrequent exacerbators identified 23 differentially expressed proteins [47]. These were associated with IgA production and immunity and phenylalanine metabolism [47]. Using a similar approach on BAL cells from ‘healthy’ smokers and patients with early stage COPD, dysregulation of several phagocytosis-related pathways was indicated particularly in females [48]. These proteomic changes and pathways also correlated to lung function (FEV1/FVC, FcγR-mediated phagocytosis), disease severity (FEV1, actin cytoskeleton) and emphysema (CT <-950 Hounsfield Units (HU), lysosomal pathway) in women [48].

Future studies in this area will include analysis of transcriptome datasets from the large and small airways as well as the alveoli to define drug action and efficacy. These can identify clusters of genes that select “drug target” signatures that enable drug repositioning in different diseases [49]. It is likely that this approach will be increasingly used in COPD as demonstrated by effective identification of infliximab signatures in sarcoidosis [49].

### Airway and lung structural cells

Transcriptomic analysis of cultured HBECs was the first site to clearly demonstrate abnormal expression of redox genes in COPD [50]. Later, the Nrf2 expression and that of its downstream target genes was found to be significantly higher in the bronchial epithelium of COPD patients who were active smokers compared to ex-smokers [51]. In contrast, these genes were not differentially expressed by smoking in alveolar macrophages suggesting a greater susceptibility of the airway epithelial cells to cigarette smoking effects. Moreover, RNA sequencing has been used on human bronchial epithelial cells from five healthy donors to identify key pathways linking exposure to cigarette smoke and cellular senescence [52] (Fig. 2). 1534 DEGs were enriched in pathways associated with reactive oxygen species, proteasome degradation and NF-κB signalling providing a molecular link between cigarette smoke exposure and senescence of airway epithelial cells [52].

Machine learning approaches are useful in gaining greater understanding of complex and heterogeneous diseases such as COPD [53]. The levels of 15 genes are commonly affected in HBECs exposed to cigarette smoke, DNA damage, oxidative stress or inflammation [53] (Fig. 2). The 8 up-regulated genes included thioredoxin-binding protein (TXNIP) that attenuates thioredoxin (TRX) antioxidant functions and causes ROS accumulation [53]. The expression levels of these 15 genes differentiated smokers and COPD subjects from non-smokers and a “potential risk factor” index was able to give a quantitative risk score for COPD in subjects.

Gene expression data from analysis of small airway epithelial cell transcriptomes of COPD patients and cigarette smoking revealed 38 up- and 114 down-regulated genes in COPD compared with healthy non-smoking controls [54]. The up-regulated genes included the inflammatory genes IL-1β, CCL2, CCL23 and CXCL14 with IL-1β being the most closely linked to the COPD disease trait by WGCNA. Furthermore, raised IL-1β mRNA expression was only observed in epithelial cells of the small airways of COPD patients and not in the blood, lung tissue or sputum of COPD patients [54].

RNA sequencing analysis of human airway smooth muscle cells identified the neutrophil-promoting cytokine CSF3 as being synergistically induced by IL-17 A and dexamethasone [55]. In a mouse COPD model, dexamethasone upregulated CSF3 expression and did not alleviate neutrophilic airway inflammation and pathology. IL-17 A or CSF3 inhibition restored dexamethasone sensitivity in this model suggesting a potential treatment for steroid resistant neutrophilic airway inflammation in COPD [55].

Rhinoviral infection of HBECs from COPD subjects enhanced SOD1 and SOD2 expression [56]. In addition, although antioxidant responses in COPD HBECs were unaffected by H_2_O_2_, H_2_O_2_ reduced the induction of anti-viral genes such as IFNβ by poly(I:C) in a TLR3-dependent manner [56]. Furthermore, H_2_O_2_ potentiates basal and rhinovirus-stimulated IL-33 expression by NHBE cells and NAC significantly attenuated basal IL-33 expression from COPD but not healthy HBECs indicating an enhanced oxidative drive in COPD cells [57]. Systemic administration or overexpression of TRX protects against viral infection and against mouse models of COPD by reducing reactive oxygen species and thereby blocking inflammation [58]. The mechanism for these effects of TRX are distinct from those of corticosteroids.

The expression of the oxidant-generating NADPH oxidase homolog, dual oxidase 1 (DUOX1) is reduced in COPD small airways and this correlated with the decline in lung function and in remodelling of the small airways and alveoli [59]. These associations were confirmed in a murine model of COPD. Thus, suppression of DUOX1 in the small airway epithelia may contribute to COPD pathogenesis [59].

The cytosolic ωGST, GSTO1-1, plays a role in glutathionylation and a specific inhibitor of mammalian GSTO1-1, ML175, inhibits lipopolysaccharide (LPS)-stimulated inflammation [60]. S-glutathionylation of reactive protein cysteines is reversed by glutaredoxin (GLRX), a deglutathionylating enzyme. In addition, ablation of GLRX enhances lung and airway fibrosis through an action on airway epithelial basal stem cells [61].

ATII cells act as stem cells within the alveoli to maintain and repair lung tissues [62]. Prolonged cigarette smoke exposure of mice induced emphysematous changes and also increased ATII stem cell numbers with a high capacity to form colonies and resist apoptosis. Transcriptomic analysis revealed upregulation of pathways related to inflammation and the circadian rhythm [62]. Interestingly, the impact of oxidative stress in cigarette smoke exposed mice is affected by the circadian clock resulting in enhanced lung inflammation and COPD-like pathology via a sirtuin 1 (SIRT1)-BMAL1 pathway [63].

The receptor for advanced glycation end products (RAGE) has been proposed as a biomarker for COPD susceptibility or progression and its expression is elevated in alveolar epithelial cells [64]. Cigarette smoke extract exposure of human A549 cells enhanced ROS/RNS generation, impaired the antioxidant responses and elevated pro-inflammatory mediator release [64]. RAGE inhibition reduced cigarette smoke-enhanced A549 cell ROS/RNS production and inflammation through effects on the Nrf2 pathway highlighting the importance of RAGE, ROS and Nrf2 in maintaining alveolar epithelial integrity [64]. RAGE also mediates pulmonary oxidative stress (4-hydroxynonenal, 4-HNE), activation of alveolar macrophages and emphysema following exposure to cigarette smoke via the Nrf2 and endoplasmic reticulum stress pathways in C57BL/6 mice [65].

Epithelial mesenchymal transition (EMT) occurs in COPD and this process is regulated by cigarette smoke-mediated changes in CD147 expression in an oxidative stress-dependent manner [66]. Similarly, the enhanced autophagy seen in COPD may result from the ability of cigarette smoke extract to enhance the expression of the fork head box class O (FOXO)1 transcription factor, autophagy-related proteins and inflammatory mediator release from ATII-like A549 cells [67].

Features of accelerated ageing including cellular senescence, DNA damage, oxidative stress and ECM remodelling are evident in COPD [68] (Fig. 2). Lung fibroblasts from subjects with severe, early-onset (SEO)-COPD and older COPD subjects were assessed at baseline and after exposure to paraquat to induce a senescent phenotype [68]. COPD fibroblasts had elevated numbers of cells staining for senescence-associated β-galactosidase (SA-β-gal), p16 expression, DNA damage and oxidative stress compared with control fibroblasts and similar changes were seen in cells from SEO-COPD subjects [68]. ECM changes were more prevalent following paraquat treatment. These data highlight the importance of cellular senescence, DNA damage and oxidative stress in the development of COPD together with a possible link to ECM dysregulation [68].

### Bronchoalveolar lavage and sputum analysis

Few studies have measured transcriptomic data from BAL cells. Meta-analysis of the transcriptomic profiles of macrophage models stimulated for various times by LPS, LPS and interferon-γ, IFNγ, IL-4, IL-10 or dexamethasone identified alveolar macrophages as having a high similarity to IL-10 activated cells with a reduced enrichment of the IFNγ-stimulated macrophage gene signature in COPD [69].

Induced sputum levels of malondialdehyde (MDA), 8-isoprostane, nitrotyrosines, and 8-oxodG are enhanced whilst BAL levels of nitrotyrosine and of reduced glutathione (GSH) being raised and lowered respectively [35]. The induced sputum expression profiles of GSH, MPO, neutrophil elastase (NE), SOD and 8-iso-PGF2α has been examined in 20 COPD patients [70]. A significant reduction in the glycerophospholipid metabolism pathway was seen in severe versus moderate COPD. This pathway is important in the regulation of redox stress and the expression of glycerophospholipid metabolites was significantly negatively correlated with SOD, MPO and 8-iso-PGF2α [70].

Pulmonary function significantly correlated with decreased sputum peroxynitrite inhibitory activity in patients with COPD [7]. Various indicators of protein nitration had elevated levels in the BALF of patients with severe asthma. These included levels of 3-nitrotyrosine, 3-bromotyrosine, and 3-chlorotyrosine. In addition, COPD bronchi express greater levels of nitrotyrosine and MPO as severity increased [7]. These data suggest that ROS and RNS are associated with the enhanced lung inflammation seen in COPD and that novel delivery of antioxidants or denitration agents are likely to provide meaningful therapeutic effects in patients with COPD.

### Peripheral blood analysis

WGCNA on peripheral blood transcriptomes from 238 COPD subjects discovered 17 modules of co-expressed genes associated with FEV1 and 3 modules were replicated in a validation cohort of 381 subjects [71]. Two modules correlated negatively with FEV1 and were enriched in neutrophil gene expression including IL-8 and IL-10 pathways. The other module correlated strongly with CD4+, CD8 + T cell-specific gene expression together with pathways related to DNA transcription and translation [71]. In addition, reanalysis of 7 publicly available blood microarray expression datasets from COPD patients identified 3,315 statistically significant DEGs and key pathways associated with Wnt signaling, cytokine-cytokine interactions, PI3K and MAPK signalling [72] (Fig. 2).

Current smoking is a confounder in many omics analysis of COPD where the effect of active smoking often exceeds that of disease [73]. Accurate assessment of current smoking habits is therefore crucial. The combination of exhaled carbon monoxide and self-reported smoking provides a better indication of active smoking and enables the delineation of an improved smoking-related blood gene expression signature in COPD [74]. In this study, 4 peripheral blood genes were identified (LRRN3, PID1, FUCA1, GPR15) which were associated with metabolic processes, pathways linked to hormonal stimuli and responses to hypoxia [74].

WGCNA analysis of blood transcriptomes in the Treatment of Emphysema With a Gamma-Selective Retinoid Agonist (TESRA) study identified a module and genes significantly associated with COPD exacerbations [75]. The key genes were linked to B and NK cell activation and to viral infection. In addition, the level of MPO in plasma was correlated with recent exacerbation rate. The data indicates that a blood gene signature of COPD exacerbation can persist for many months after the attack [75].

WGCNA also identified a 120 gene module from peripheral blood mRNA and miRNA that was able to discriminate between pneumonia and COPD exacerbations [76]. The authors identified hepatocyte nuclear factor 4α (HNF4A), mutated in colorectal cancer (MCC) and mucin 1 (MUC1) as central network regulators which, together with miR-545-3p and miR-519c-3p, distinguished COPD exacerbations from community acquired pneumonia [76].

Proteomics analysis of plasma from 43 mild/moderate COPD patients and 43 age- and sex-matched controls, identified 20 differentially expressed proteins involved in inflammation including haptoglobin, the blood coagulation and complement pathway, oxidative stress and lipoprotein/lipid metabolism [77]. Lipid peroxidation products, protein carbonyls and superoxide anion and total oxidative stress measures are reproducibly elevated in the blood of patients with COPD in comparison to controls and some instances show links to disease severity [35] (Fig. 2). In contrast, there have been variable reports regarding oxidative stress-damaged DNA, peroxynitrite and nitrotyrosine upregulation and down-regulation of antioxidant responses in COPD [35]. However, a recent systematic review and meta-analysis of 18 studies indicated that the blood levels of reduced GSH were significantly reduced in COPD compared with control subjects [78].

Generally, the studies report enhanced DNA damage and nitrosative stress and reduced antioxidant capacity whilst studies of antioxidant vitamins, such as A, C and E, do not give consistent results. The vitamin E isoform γ-tocotrienol protects against emphysema and improves measures of lung function in a murine cigarette smoke-induced COPD model [79]. γ-Tocotrienol also attenuated cigarette smoke-induced airway remodelling, BAL neutrophilia, inflammatory mediator expression and oxidative damage biomarkers, in part, through activation of the Nrf2 pathway [79]. These effects of γ-tocotrienol were greater than those seen with prednisolone supporting a potential role in the treatment of COPD [79]. In addition, vitamin D expression is closely linked to oxidative stress and vitamin D dysregulation is often seen in COPD patients who also have asthma [10].

In a small cross-sectional study, the peripheral blood expression of MDA and uric acid was increased whilst that of ascorbic acid/vitamin C was significantly decreased in 28 COPD patients with GOLD I/II disease compared to healthy control subjects [80]. In addition, a meta-analysis of 14 studies involving 817 COPD patients and 530 healthy controls indicated that blood MDA levels are significantly greater in COPD compared with healthy subjects [81].

GGT (gamma-glutamyltransferase) is an oxidative stress marker. In a single centre study examining serum GGT levels in 117 patients during stable COPD and AECOPD demonstrated raised serum levels in stable COPD compared with control subjects [82]. A GGT level of 21.2 IU/L gave a good diagnostic prediction of COPD. Serum levels were elevated to a greater extent in AECOPD compared to stable COPD and serum levels of 26.5 IU/L predict COPD exacerbations [82]. Moreover, ROS modulator 1 (Romo1) is involved in several oxidative stress-related diseases [83]. Serum Romo1 expression was greater in a small (49 COPD versus 34 healthy control subjects) study and its expression correlated inversely with FEV1% predicted in COPD patients and positively with serum CRP and ROS levels. The data suggest that Romo1 may be a good biomarker of ROS in COPD lungs [83].

Finally, serum total oxidant status (TOS), total antioxidant status (TAS) and of the oxidative stress index (OSI) were measured in 137 patients with COPD and 102 healthy individuals [84]. Oxidant status levels were highest in the current smoking compared to ex-smoking COPD group and never smoking COPD group. TOS was particularly associated with smoking status rather than with COPD and TAS was significantly reduced in actively smoking COPD subjects compared to healthy smokers [84]. However, there was no difference in OSI between groups suggesting that pathogenetic mechanisms beyond oxidant/antioxidant balance are important in smoking-related COPD [84].

### Cross-tissue validation

As samples from the airways and lungs of COPD patients are difficult to obtain it is important to understand how biomarker and gene expression profiles in a readily accessible tissue compartment such as blood relates to those at the site of disease. RNA-sequencing of large-airway epithelial cells, alveolar macrophages and peripheral blood from a subset of COPD and healthy subjects from the COPDGene study has been performed [85]. Examination of links between DEGs in all samples with lung function, emphysema, airways disease and smoking history revealed no specific DEGs across all three sample types [85]. As expected, CYP1B1 and AHRR, both cigarette smoke exposure-related genes, were differentially expressed in the large-airway epithelium according to smoking status. Overall, expression in epithelial cells and macrophages correlated significantly with smoking and airway disease phenotypes [85]. However, emphysema was associated with enrichment of pathways involved in hemostasis and immune signalling across tissue samples. Emphysema was also linked to genes associated with B cell function in peripheral blood [85].

WGCNA of blood, lung tissue and sputum from mild-moderate COPD patients and healthy controls showed that differences in macrophage infiltration, smoking status or airflow limitation were associated with modules enriched for pathways linked to iron transport, extracellular matrix and cilium organization [86]. These changes in the lung did not translate well to blood.

A meta-analysis of transcriptomes from COPD lung tissue, blood and sputum has also been performed using WGCNA on previously published datasets [87]. 21 WGCNA modules were identified in one lung tissue analysis that was replicated (86%) in an independent lung tissue cohort but less so in sputum (33%) and blood (23%) [87]. Three modules that were associated with airflow limitation were maintained across sputum lung samples, but not blood, and included genes associated with mitochondria, iron homeostasis, RNA processing and T cells. The data suggests that mitochondrial dysfunction is important is COPD pathogenesis and that blood transcriptomics differentiate between the lung and systemic aspects of COPD [87].

However, the use of an R package, MetaCorrelator, that identifies reproducible transcriptional signatures correlated with disease phenotypes across multiple datasets, [88] revealed a gene expression profile that predicted lung function in COPD patients in both blood and tissue samples. These genes related to dysregulated oxidation status together with altered inflammatory states [88].

### Multi-omics integration

The combination or integration of several types of omics data should provide greater clarity regarding pathways and processes that drive disease and may also be suitable for improving the accuracy of COPD molecular classification. The first study to undertake such an analysis in COPD utilised 9 different omics datasets (mRNA, miRNA, proteomes and metabolomes) across different sites from 52 female subjects. Data was integrated using a bioinformatic tool known as similarity network fusion (SNF) [89]. The authors reported improved accuracy in the ability to differentiate healthy never-smokers and healthy smokers from COPD. There was also an important increase in the power to differentiate with a reduction in necessary group sizes from 30 to 6 [89]. Overall, the results suggested that integrating five to six data sets enables small cohorts to be accurately defined using unsupervised molecular classification.

A deeper analysis integrating transcriptomic data with GWAS data has also been performed with the goal of confirming GWAS data and revealing novel candidate causal genes [90]. This analysis included a lung expression quantitative trait locus (eQTL) dataset from 1038 individuals and 13,710 cases and 38,062 controls from the International COPD Genetics Consortium (ICGC) and validation using lung data from the GTEx project [90]. Twelve new candidate genes or loci were identified for COPD and replicated in GTEx and included myotonic dystrophy protein kinase (DMPK) which is linked to antioxidant capacity. Further analysis of this type may enable clearer translation of COPD genetics into the clinic [90].

Transcriptomics and metabolomics from the blood of 149 current or former smokers with or without COPD have been overlayed [91]. Glycerophospholipid metabolism was associated with the number of COPD exacerbations and worse airflow obstruction, whilst severe exacerbations requiring hospitalisation were linked to sphingolipid metabolism [91]. In addition, lung function was associated with fat digestion and absorption and T cell receptor signaling and emphysema was associated with oxidative phosphorylation [91]. Overall, metabolomic studies have highlighted defects in amino acid metabolism, energy production, lipid metabolism and an oxidant/antioxidant dysregulation in COPD which has been linked to local and systematic NF-κB-driven inflammation [92].

Integration of gene expression profiles with GWAS analysis using expression data from lung tissue and blood has been correlated with severe COPD and emphysema [93]. Seven genes were significantly associated with severe COPD whilst emphysema was significantly linked with five genes in blood or lung tissue. Signals for PSMA4 (proteasome 20 S subunit Alpha 4), WNT3 (Wnt Family Member 3), DCBLD1 (Discoidin, CUB And LCCL Domain Containing 1), LILRA3 (Leukocyte Immunoglobulin Like Receptor A3) and EGLN2 (Egl-9 Family Hypoxia Inducible Factor 2) were validated in a separate cohort providing tissue selective validation of COPD susceptibility loci [93].

An interesting study has also examined the gene expression profile in the quadriceps muscle of 79 stable COPD subjects and 16 healthy controls [94]. Skeletal muscle dysfunction in COPD occurs frequently and this influences the patient’s quality of life (QoL) and survival. 1,826 DEGS were reported between healthy and COPD quadriceps muscle of which 31 transcripts, including family with sequence similarity 13 member A (FAM13A), were which has been linked to COPD previously through genome-wide association [94]. Network co-expression analysis identified 6 modules including one with mitochondrial and extracellular matrix pathway features and one representing proliferation and differentiation [94].

Pulmonary rehabilitation is the only effective treatment for COPD and in a cigarette smoke exposed mouse model, exercise limits the induction of emphysema via up-regulation of Nrf2 and HO-1 and a reduction in inflammation [95]. This effect was mediated by the release of irisin, a myokine secreted from the muscle during exercise, which is known to stimulate Nrf2. In addition, salidroside is a glucoside of tyrosol found in the plant Rhodiola rosea and in cigarette smoke-exposed rats reduces both the emphysema scores and skeletal muscle atrophy acting by down-regulating myostatin expression and enhancing myogenin levels [95].

Cultured myoblasts and myotubes from the quadriceps of COPD patients demonstrate enhanced autophagy compared to healthy subjects and this was prevented by the antioxidant ascorbic acid/vitamin C [96]. This data indicates the importance of systemic oxidative stress in COPD myotube atrophy in vitro [96]. In differentiated myotubes cultured from muscle biopsies of patients with COPD cachexia, the PDE-4 inhibitor roflumilast upregulated the Nrf2 pathway and the expression of its downstream antioxidant sirtuin-1 [97]. The use of three-dimensional (3D) cell models has potential use in COPD research. Cigarette smoke exposure of 3D human bronchial epithelial cell organoids modulated central carbon metabolism, oxidative stress responses and heightened the release of mucins and MMPs reflecting pathophysiological changes in COPD airways [98]. These 3D organoids may provide novel insights into COPD pathogenesis and act as a good model for drug discovery [98].

Several papers have reported similarities and differences between transcriptomic analysis of COPD compared to other lung diseases particularly idiopathic pulmonary fibrosis (IPF) and lung cancer. For example, Maghsoudloo and colleagues reported that the asthma lung transcriptome was more similar to COPD than IPF [99]. There were 1,907 DEGs found overall between COPD lung tissue and that from IPF and asthma and analysis of drug-target networks suggested potential drug therapies such as anti-CD20, anti-CD52, anti-VEGFA and anti-inflammasome candidate drugs [99]. In a similar approach using RNA-sequencing in 87 lung samples from patients with COPD, IPF or healthy controls, both emphysema and IPF DEGs indicated enrichment for the p53/hypoxia pathway [100]. Addition of miRNA data identified miR-96 as a regulatory hub for the p53/hypoxia network [100].

The presence of COPD heightens the risk of lung cancer, and it is likely that the COPD stroma possess tumourigenic mechanisms. Transcriptomic and proteomic analysis of lung samples from COPD patients with lung cancer (tumour and adjacent non-malignant tissue) and of samples from COPD patients who do not have lung cancer was integrated using the Joint and Individual Variation Explained (JIVE) method [101]. JIVE identified eight patterns which stratified tumour, adjacent and control tissues. Transcriptomic data drove the differentiation between tumour and adjacent stroma and was associated with enrichment of ECM and activation of the PI3K pathway [101].

### Mouse versus man

For translational studies and the successful transfer of pre-clinical studies into successful clinical trials, it is important to be able to compare human data to that from animal models of disease to ensure the same molecular pathways are being activated. The transcriptomes of murine lung exposed to cigarette smoke have been compared to the previously published 386 gene “human smoking” signature, the 7 gene mild COPD signature and the 4,071 gene severe COPD signature from human COPD [102]. Six-month cigarette smoke exposed mice, corresponding to the time when COPD-like physiology and morphology is present, demonstrated that 48% of the human smoking and 27% of human COPD severity genes were shared between species [102]. However, there was no enrichment of the human mild COPD signature in these mouse models [102].

In a separate study, using 4 different cigarette smoke-exposed mouse models of COPD, Yun and co-workers identified xenobiotic metabolism and Nrf2-mediated oxidative stress response pathways as common smoking response pathways in emphysema-sensitive animals [103]. Overall, despite limited sharing of individual genes across species shared pathways were prevalent. Translation of the response to therapies from mouse to man models will be improved by the use of bioinformatic tools such as Found in Translation [104].

## Conclusions

**Fig. 3:**
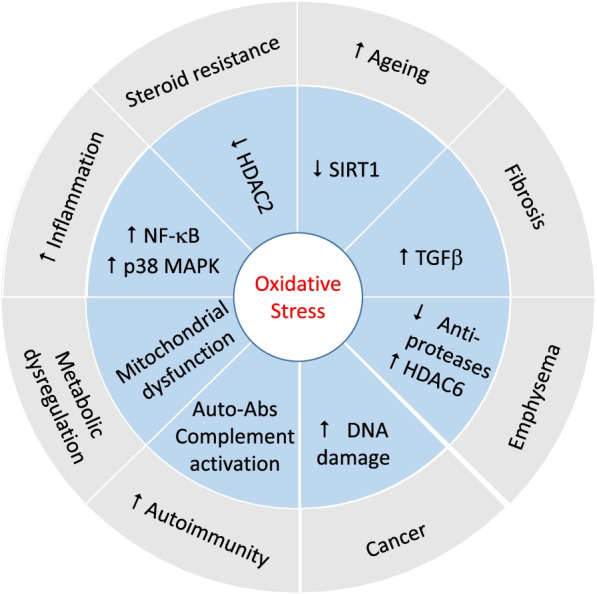
Summary of the mechanisms and effects of oxidative stress in driving COPD pathophysiology. Abbreviations: NF-κB = nuclear factor κB; p38 MAPK = p38 mitogen activated protein kinase; TGFβ = transforming growth factor β; SIRT1 = sirtuin 1; HDAC = histone deacetylase

There is a plethora of data gathered over many years indicating enhanced levels of oxidative stress in COPD. This dysregulated oxidant/antioxidant balance is considered to be an important driver of COPD pathogenesis as oxidative stress drives most of the key features of COPD including inflammation, emphysema, small airway fibrosis, mucus hyperplasia as well as auto-antibody production, metabolic dysregulation, ageing and relative corticosteroid refractoriness (Fig. 3) making this an important therapeutic target. The development of novel antioxidant therapies should improve all aspects of COPD but results of clinical trials to date have been disappointing. This may reflect a poor local antioxidant effect provided by oral drugs and new inhaled delivery approaches are being tested. In addition, other approaches such as the use of liposomes and nanoparticles to deliver antioxidants to specific cell types have been developed. It is also important to have good validated biomarkers of local antioxidant effects to ensure good drug coverage. It is essential to perform additional research in this area.

## Data Availability

Data are available in public databases or in published papers.

## Abbreviations

•OH: Hydroxyl radicals
3D: Three-dimensional
4-HNE: 4-hydroxynonenal
AECOPD: Acute exacerbations of COPD
AP-1: Activator protein 1
ASM: Airway smooth muscle
ATII: Type II alveolar epithelial cell
BAL: Bronchoalveolar lavage
BALF: Bronchoalveolar lavage fluid
CFTR: Cystic fibrosis transmembrane conductance regulator
CHRM3: Cholinergic receptor muscarinic 3
COPD: Chronic obstructive pulmonary disease
CVD: Cardiovascular disease
DCBLD1: Discoidin, CUB And LCCL Domain Containing 1
DEGs: Differentially expressed genes
DMPK: Myotonic dystrophy protein kinase
DUOX1: Dual oxidase 1
ECM: Extracellular matrix ()
EGLN2: Egl-9 Family Hypoxia Inducible Factor 2
EMT: Epithelial mesenchymal transition
eQTL: Expression quantitative trait locus
ETC: Electron transport chain
EvA: Emphysema versus airways disease project
FAM13A: Family with sequence similarity 13 member A
FAO: Fatty acid oxidation
Fe: Iron
FEV1: Forced expiratory volume in 1 second
FIS1: Fission, Mitochondrial 1
FOXO1: Fork head box class O 1
GGT: Gamma-glutamyltransferase
GLRX: Glutaredoxin
GOLD: Global initiative for chronic obstructive lung disease
GPx: Glutathione peroxidase
GSH: Reduced glutathione
GSTs: Glutathione S-transferases
GTEx: Genotype-Tissue Expression database
GWAS: Genome-wide association study
H_2_O_2_: Hydrogen peroxide
HDAC: Histone deacetylase
HNF4A: Hepatocyte nuclear factor 4α
HO-1: Heme oxygenase-1
HU: Hounsfield Units
IPF: Idiopathic pulmonary fibrosis
IREBP2: Iron responsive element binding protein 2
JIVE: Joint and Individual Variation Explained
LILRA3: Leukocyte Immunoglobulin Like Receptor A3
LPS: Lipopolysaccharide
MCC: Mutated in colorectal cancer
MDA: Malondialdehyde
MDMs: Monocyte-derived macrophages
MFN1: Mitofusin 1
MIRO1: Mitochondrial Rho GTPase 1
MPO: Myeloperoxidase
MSCs: Mesenchymal stem cells
mtROS: Mitochondrial reactive oxygen species
MUC1: Mucin 1
NE: Neutrophil elastase
NO2: Nitrite
Nrf2: Nuclear factor erythroid 2-related factor 2
O^-^_2_: Superoxide anions
ONOO: Peroxynitrite
OPA1: Optic atrophy 1
OSI: Oxidative stress index
OXPHOS: Oxidative phosphorylation
PC: Protein carbonylation
p-DRP1: Phosphorylated dynamin-related protein 1
PGAM5: Phosphoglycerate mutase family member 5
PI3K: Phosphoinositide-3-kinase ()
PINK1: PTEN Induced Kinase 1
PSMA4: Proteasome 20S subunit Alpha 4
PTEN: Phosphatase and tensin homolog
QoL: Quality of life
RAGE: Receptor for advanced glycation end products
RNS: Related nitrogen species
ROS: Reactive oxygen species
SA-β-gal: Senescence-associated β-galactosidase
SEO: Severe, early-onset
SIRT1: Sirtuin 1
SOD2: Mitochondrial superoxide dismutase
SPIROMICS: Subpopulations and Intermediate Outcomes in COPD Study
sTfR: Soluble transferrin receptor
TAS: Total antioxidant status
TESRA: Treatment of Emphysema With a Gamma-Selective Retinoid Agonist
TGFβ: Transforming growth factor-β
TLCOC: Carbon monoxide transfer factor
TOS: Total oxidant status
TRX: Thioredoxin
TXNIP: Thioredoxin-binding protein
WGCNA: Weighted Gene Co-expression Network Analysis
WNT3: Wnt Family Member 3
Wnt: Wingless-related integration site
ΔΨm: Mitochondrial membrane potential
